# High-Level Carbapenem Resistance among OXA-48-Producing *Klebsiella* *pneumoniae* with Functional OmpK36 Alterations: Maintenance of Ceftazidime/Avibactam Susceptibility

**DOI:** 10.3390/antibiotics10101174

**Published:** 2021-09-27

**Authors:** Pilar Lumbreras-Iglesias, María Rosario Rodicio, Pablo Valledor, Tomás Suárez-Zarracina, Javier Fernández

**Affiliations:** 1 Department of Clinical Microbiology, Hospital Universitario Central de Asturias, 33011 Oviedo, Spain; pilar.lumbreras08@gmail.com; 2 Traslational Microbiology Group, Instituto de Investigación Sanitaria del Principado de Asturias (ISPA), 33011 Oviedo, Spain; rrodicio@uniovi.es; 3 Department of Functional Biology, Microbiology Area, University of Oviedo, 33006 Oviedo, Spain; 4 Research & Innovation, Artificial Intelligence and Statistical Department, Pragmatech AI Solutions, 33003 Oviedo, Spain; pablo.valledor@pragmatech.ai; 5 Department of Internal Medicine, Hospital Universitario Central de Asturias, 33011 Oviedo, Spain; tomassecades@hotmail.com

**Keywords:** *K. pneumoniae*, carbapenem resistance, OXA-48, porins, ceftazidime/avibactam

## Abstract

The aim of this work was to analyze outer membrane porin-encoding genes (*ompK35* and *ompK36*) in a collection of OXA-48 producing *Klebsiella pneumoniae*, to assess the effect of porin alterations on the susceptibility to ceftazidime/avibactam, and to describe a screening methodology for phenotypic detection of OXA-48-producing *K. pneumoniae* with disrupted porins. Antimicrobial susceptibility was tested by Microscan and Etest. The genomes of 81 OXA-48-producing *K. pneumoniae* were sequenced. MLST, detection of antimicrobial resistance genes, and analysis of *ompK35* and *ompK36* were performed *in silico*. Tridimensional structures of the OmpK36 variants were assessed. Receiver operating characteristics curves were built to visualize the performance ability of a disk diffusion assay using carbapenems and cefoxitin to detect OmpK36 functional alterations. A wide variety of OmpK36 alterations were detected in 17 OXA-48-producing *K. pneumoniae* isolates. All displayed a high-level meropenem resistance (MIC ≥ 8 mg/L), and some belonged to high-risk clones, such as ST15 and ST147. Alterations in *ompK35* were also observed, but they did not correlate with high-level meropenem resistance. All isolates were susceptible to ceftazidime/avibactam and porin alterations did not affect the MICs of the latter combination. Cefoxitin together with ertapenem/meropenem low inhibition zone diameters (equal or lower than 16 mm) could strongly suggest alterations affecting OmpK36 in OXA-48-producing *K. pneumoniae*. OXA-48-producing *K. pneumoniae* with porin disruptions are a cause of concern; ceftazidime/avibactam showed good *in vitro* activity against them, so this combination could be positioned as the choice therapy to combat the infections caused by this difficult-to-treat isolates.

## 1. Introduction

The use of carbapenems has been increasing in hospitals in the past years as a consequence of the rise of extended spectrum β-lactamase (ESBL) and/or AmpC-producing *Enterobacterales* [1]. The extensive clinical use of these drugs has led in turn to the emergence of carbapenem-resistant *Enterobacterales* (CRE), boosted by the spread of carbapenemases, such as serine β-lactamases (KPC and OXA-48) or metallo-β-lactamases (VIM, IMP and NDM), by means of mobile genetic elements [2]. Carbapenem resistance can also be mediated by permeability defects in the outer membrane of ESBL- or AmpC-producing strains [3], which can besides increase the minimal inhibitory concentrations (MICs) of carbapenems in carbapenemase-producing *Enterobacterales* (CPE). These defects can be associated with alterations in porin-encoding genes or their promoter regions, such as point mutations, deletions or insertions which can hinder the synthesis of a functional protein, hence obstructing the entry of molecules into the periplasm [4]. *Klebsiella pneumoniae* is one of the main reservoirs of carbapenemases in the hospital environment [5]. OmpK35 and OmpK36 are the major porins in this species, and they constitute the main pathway through which carbapenems enter the cell [6].

The increasing carbapenem resistance among *Enterobacterales* poses a major threat to public health because of its difficult treatment. Several clinical studies have demonstrated that carbapenems, alone or combined with other drugs, are options for the treatment of infections caused by CRE when MICs of these compounds are low [7,8]. This is the case of most infections caused by OXA-48-producing *Enterobacterales*, which weakly hydrolyze carbapenems [9,10]. However, when MICs of meropenem are higher than 8 mg/L, the probability of reaching therapeutic success is low according to stochastic modeling data, and thus carbapenems are contraindicated [7]. In response to the emergence and spread of CRE, new antimicrobials or combinations of a β-lactam plus a β-lactamase inhibitor are either under development or have been approved in the last years. This is the case of ceftazidime/avibactam, a combination of a third-generation cephalosporin with a synthetic β-lactamase inhibitor that prevents the activities of Ambler class A and C β-lactamases and some Ambler class D enzymes including OXA-48 [11].

In view of the worrisome medical situation raised by CRE, and the recognized contribution of porin alterations to carbapenem resistance, the aims of the present study were (i) to molecularly analyze outer membrane porin-encoding genes in a collection of OXA-48 producing *K. pneumoniae* isolates; (ii) to assess the effect of the observed alterations on the susceptibility to ceftazidime/avibactam; (iii) to develop a screening methodology for phenotypic detection of OXA-48 producing *Klebsiella* spp. with porin disruptions.

## 2. Results and Discussion

### 2.1. Carbapenems and Ceftazidime/Avibactam Susceptibility, β-Lactamases and Molecular Epidemiology

Bacterial identification by MALDI-TOF recognized the 81 isolates as *K. pneumoniae*, results which were confirmed *in silico* by analysis of the sequenced genomes. Strains of *K. pneumoniae* were classified in 15 sequence types (ST), being ST326 (22/81, 27.2%) and ST147 (20/81, 24.7%) the most prevalent (Table 1 and Table S1). The presence of *bla*_OXA-48_ was confirmed in all of them. Antimicrobial susceptibility testing, performed with the Microscan system, showed that 100%, 24.69% and 20.99% isolates were resistant to ertapenem, imipenem and meropenem, respectively. In addition to the five isolates selected from other samples, twelve recovered from blood cultures displayed high level of meropenem resistance (MIC ≥ 8 mg/L), which could not be explained only by the OXA-48 production. This suggested the existence of additional mechanisms. Apart from β-lactamase production, mechanisms which can lead to a high-level carbapenem resistance comprise efflux pumps, PBP (penicillin binding protein) alterations, and mutations that modify the expression and/or function of outer membrane porins, the latter being a mechanism frequently found in *Enterobacterales* [6]. All isolates of the study were susceptible to ceftazidime/avibactam, including those highly resistant to meropenem, with MICs ranging from 0.19 mg/L to 0.75 mg/L (three of the latter did not coproduced ESBLs and were also susceptible to ceftazidime alone). Relevant features of the 17 isolates displaying high-level carbapenem resistance are shown in Table 1, while the same features relative to the remaining isolates are compiled in Supplementary Table S2.

Moreover, 82% of the isolates displaying high-level carbapenem resistance (14/17) coproduced ESBLs, with roughly the same rate (52/64, 81%) found among the isolates with low carbapenem MICs (MICs ≤ 4 mg/L). All of the isolates which did not coproduce ESBLs were susceptible to ceftazidime alone, including those three that displayed high-level carbapenem resistance. *bla*_SHV-12_ and *bla*_CTX-M-15_ were the most frequent genes detected among the isolates that showed an ESBL phenotype (Table 1 and Table S1). Based on the antimicrobial susceptibility results, and considering that three of the highly-carbapenem resistant isolates did not co-produce ESBLs, there does not appear to be a necessary correlation between production of these enzymes and high MICs of meropenem. For this reason, a detailed analysis of the genes encoding the main porins of *K. pneumoniae*, OmpK35 and OmpK36, and their upstream regions was performed, in order to infer possible permeability alterations in the outer cell membrane.

### 2.2. Molecular Analysis of OmpK35 and OmpK36 in OXA-48 Producers

Bioinformatic analysis of *ompK35* from the 17 isolates with high-level carbapenem resistance revealed a single nucleotide insertion (T) at 623 position in four of them, and a single nucleotide deletion (G) at 185 position in another one (Table 1). Both alterations led to a change in the reading frame. On the other hand, analysis of the *ompK35* sequences of the isolates exhibiting low meropenem MICs showed that three of them carried a IS*1*-like element inserted upstream of the gene, one had a G890A transition in the coding region, which resulted in a premature stop codon, and another one had a single nucleotide deletion (G) at position 575 (Table S1). Accordingly, it seems that at least not all the changes detected in *ompK35* are enough to substantially increase carbapenem MICs (Table S1). These results are in line with previous observations by other authors, reporting that alterations affecting *ompK35* are not capable to generate high resistance to carbapenems [12,13,14].

**Figure 1:**
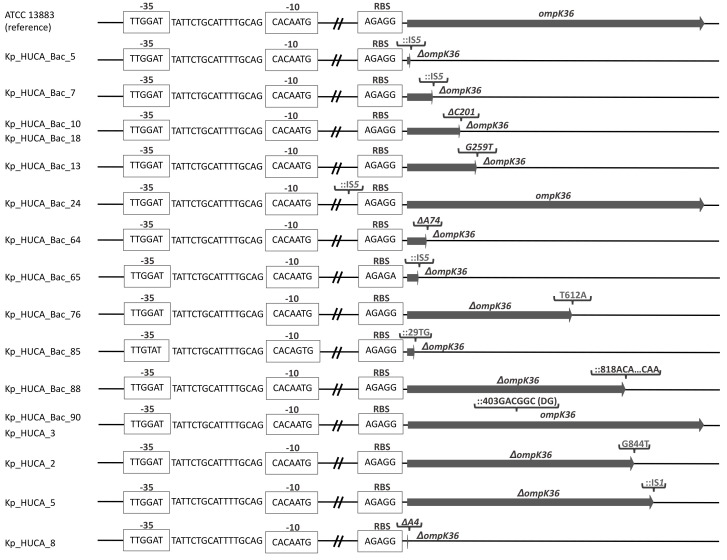
Genetic alterations in *ompK36* and upstream DNA found in OXA-48-producing *Klebsiella pneumoniae* isolates displaying high-level resistance to meropenem. Kp_HUCA_4 is not represented as it carries a large deletion that includes the promoter region and the first 702 nucleotides of the ORF. RBS, ribosome binding site; IS, insertion sequence;, insertion; Δ, deletion.

Regarding OmpK36, functional alterations have been broadly reported among KPC-producing *K. pneumoniae* [15,16,17]; however, reports and analysis of porin disruptions among OXA-48 producers are still scarce [18]. In the present study, all isolates displaying high meropenem MICs, including the 12 strains recovered from blood cultures and the five strains selected from other samples, had alterations affecting the OmpK36 coding region or the upstream DNA, which could lead to non-functional proteins or hinder the expression of the gene (Table 1 and Figure 1). The remaining isolates carried no mutations or had nucleotide changes considered as polymorphisms.

Among high-level carbapenem resistant isolates, four contained an IS*5*-like element located within the coding region (three isolates) or immediately upstream of the RSB (ribosome binding site; one isolate) of OmpK36; the latter alteration was similar to one previously reported in KPC-2-producing *K. pneumoniae* ST258, where it was shown to be associated with significantly lower OmpK36 expression levels and increased MICs of carbapenems [15]. A single isolate carried an IS*1*-like insertion within OmpK36. Additional alterations included: (i) nonsense mutations that generate premature stop codons yielding truncated and probably non-functional proteins (3 isolates); (ii) single nucleotide deletions that led to frameshift mutations (4 isolates, with two of them, both ST326, having the same deletion at the same position); (iii) a large deletion of 886 nucleotides including the first 702 nucleotides of the ORF and 184 of the upstream DNA (1 isolate; not shown in Figure 1); (iv) insertions of 2 and 34 nucleotides at positions 29 and 818, respectively, which resulted on a frame shift (each displayed by a single isolate); (v) insertion of 6 nucleotides at position 403, resulting in the incorporation of two additional amino acids (DG) on a highly conserved region (L3) of the porin (two isolates). The same insertion was associated with carbapenem resistance in KPC-2-producing *K. pneumoniae* ST423 and ST11 isolates [16]. In addition, Lunha et al. reported a very similar amino acid insertion (GD) at the same position within the L3 region of OmpK36 in ST37 and ST11 OXA-48-producing *K. pneumoniae*, and linked this alteration to an increase in the carbapenem resistance level. Such resistance increase was also observed in KPC-2-producing *K. pneumoniae* ST258 strains with the same GD insertion in OmpK36, and attributed to a diminishment in the pore diameter caused by the two additional amino acids [17].

The tridimensional structures of OmpK36 variant proteins predicted by the Phyre2 web portal are shown in Figure S1. In most of them, it can be visually verified how the conformation of the porin is affected by the observed alterations.

Our series represent a wide variety of OmpK36 alterations detected among different clones of OXA-48-producing *K. pneumoniae*. Some of them, such as ST15 or ST147, are considered of high-risk since they are clones which have a special ability for successful expansion, and are typically involved in outbreaks [19,20], which poses an additional concern. OmpK36 disruptions described in our work are probably responsible for the high MICs of meropenem of the strains in which they were detected, in comparison with MICs of strains with functional OmpK36 proteins. The increase of meropenem MICs in OXA-48 producers is especially marked since, as previously indicated, this enzyme by itself only causes weak carbapenem hydrolysis and a consequent low level of resistance to these drugs [9,10]. Carbapenems must first penetrate the outer membrane in order to reach the PBPs and, because these drugs are relatively hydrophilic, their entry occurs through the water-filled porin channels [21]. Obviously, their inactivation via periplasmic carbapenemases will be more effective in increasing resistance if the influx is decreased through the loss of porins [22,23]. Apart from allowing carbapenems to cross the outer membrane, OmpK36 constitutes the entry way into the cell of some nutrients and other physiological important substances; thus, the loss of this porin is not free of charge. In fact, Wong et al. recently demonstrated that OmpK36-mediated carbapenem resistance attenuates the virulence of *K. pneumoniae* ST258 [17].

Selection of isolates displaying high-level carbapenem resistance due to outer membrane alterations may be conditioned by several factors, including the selective pressure exerted by previous exposure to carbapenems of individual patients or by the hospital environment [17]. In this sense, 11 (64.7%) of the isolates with high-level carbapenem resistance in our study were recovered from patients who had been previously treated with carbapenems during the same hospital stay (Table 1). The high-level of meropenem resistance displayed by these isolates (meropenem MICs ≥ 8 mg/L), exclude these drugs as therapeutic alternatives to treat the infections they cause. In fact, as already mentioned in the introduction, PK/PD modeling has shown that the probability of reaching the target pharmacodynamic parameter is low, even if high dose and extended infusion are administered [7]. Fortunately, all isolates in the present study were susceptible to ceftazidime/avibactam and displayed low MICs of these drugs, regardless of the OmpK36 variant. This might be due to the fact that, unlike other β-lactamase inhibitors, the outer membrane porins are not the major route by which avibactam enters the periplasm [24], and it has been previously reported that ceftazidime MICs are not affected by OmpK36 alterations [14]. Wong et al., also, did not find a decrease in the susceptibility to ceftazidime/avibactam among *K. pneumoniae* ST258 with alterations affecting OmpK35 or OmpK36 [17].

### 2.3. Disk Diffusion Assay Using Carbapenems and Cefoxitin for Detection of Functional Alteration in OmpK36:

**Figure 2:**
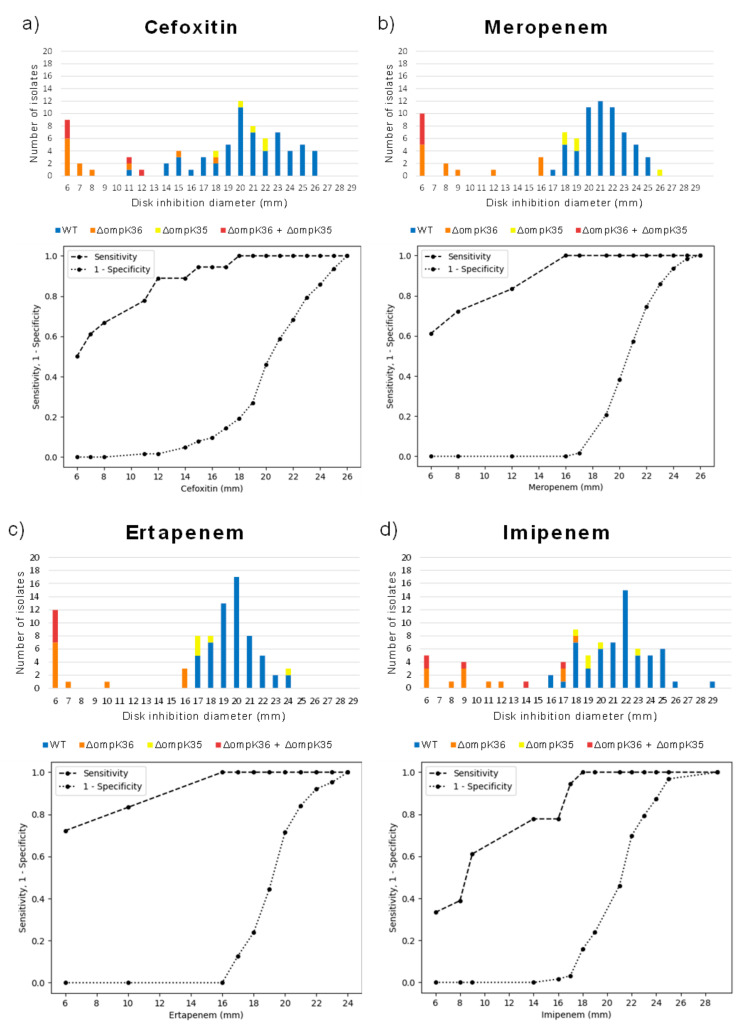
Distribution of cefoxitin 30 µg (**a**), meropenem 10 µg (**b**), ertapenem 10 µg (**c**) and imipenem 10 µg (**d**) inhibition zones (mm) of OXA-48 producing *Klebsiella pneumoniae* with and without porin alterations. Below of each graphic, a receiver operating curve (ROC), showing the evolution of sensitivity and 1-specificity to identify OmpK36 functional alterations in relation to the different thresholds applied for each antibiotic, is represented.

Detection of strains with functional alterations in porins among OXA-48-producing *K. pneumoniae* may be of particular interest in clinical microbiology laboratories, especially when affecting OmpK36. However, next-generation sequencing techniques are not available in the routine of these laboratories, so the establishment of a phenotypic screening for the detection of such strains would be very useful to predict genotypes from phenotypes and, also, for epidemiological purposes. Most of the isolates with alterations in OmpK36 displayed reduced inhibition zone diameters to cefoxitin and carbapenems. Thus, our data suggest that resistance to both drugs could be considered a surrogate marker of functional alterations in OmpK36 in OXA-48-producing *K. pneumoniae*. In addition to carbapenems, it is well known that this porin constitutes the main pathway through which cefoxitin penetrates the outer membrane [4], and alterations affecting OmpK36 have been associated with the development of resistance to this drug [25]. In order to set a threshold to detect isolates carrying functional alterations in OmpK36, a disk diffusion assay testing ertapenem, imipenem, meropenem and cefoxitin was performed, and ROC curves were built to evaluate sensitivity and specificity of their detection at different thresholds (Figure 2). All isolates displaying inhibition zone diameter lower or equal to 16 mm to both ertapenem and meropenem, carried alterations in OmpK36 with or without changes in OmpK35. However, isolates displaying higher diameters did not have alterations in the former porin. Accordingly, the sensitivity and specificity for detection of OmpK36 functional alterations was of 100% when applying this threshold. In contrast, when using an imipenem and cefoxitin disk diffusion assay, the strains with alterations in OmpK36 could not be clearly separated from those with the wild type porin (i.e., 100% sensitivity and specificity were not obtained at any threshold). Nevertheless, as mentioned above, a reduction on the inhibition zone to cefoxitin, together with an inhibition zone diameter to meropenem and ertapenem lower or equal to 16, strongly suggest alterations in this porin.

## 3. Materials and Methods

All carbapenem-resistant *K. pneumoniae* isolates recovered from blood cultures of different patients admitted to a tertiary hospital, Hospital Universitario Central de Asturias (HUCA), in northern Spain over a five-year period (2014–2019) were collected (*n* = 76). Additionally, five *K. pneumoniae* isolates with high meropenem MICs, recovered from different samples of patients admitted to the HUCA during the same period were studied. Bacterial identification was performed by MALDI-TOF/MS (Bruker Daltonics, Billerica, MA) using α-Cyano-4-hydroxycinnamic acid as a matrix and following the manufacturer instructions available on https://www.bruker.com/en/services/training, (accessed on 14 December 2020). *In silico* identification was performed by using KmerFinder, available at the Center for Genomic Epidemiology site (https://www.genomicepidemiology.org/; CGE, 2020; accessed on 25 January 2021). Antimicrobial susceptibility was determined by the Microscan system (Beckman Coulter, Brea, CA, USA) and, also, by Etest^®^ (bioMérieux, Marcy l’Etoile, France) for meropenem and ceftazidime/avibactam. Results were interpreted according to the EUCAST guidelines [26]. Screening of carbapenemases was performed by means of a previously described algorithm [27].

Genomic DNA from the 81 isolates (76 recovered from blood cultures and five from other samples) was extracted with the NZY Microbial gDNA Isolation kit (NZYTech, Lisbon, Portugal), and then sequenced by Illumina technology to generate 125 bp paired-end reads in a HiSeq 1500. Quality control of the reads was performed using FastQC software (Babraham Bioinformatics, Cambridgeshire, UK) and de novo assembly was carried out with VelvetOptimizer [28].

Multi-locus sequence types and the presence of resistance genes were determined *in silico* by the use of the MLST 2.0 and ResFinder 3.2 tools, respectively [29]. The sequences of the two major porin-encoding genes of *Klebsiella* (*ompK*35 and *ompK*36), including their upstream regions, were analyzed by bioinformatic tools, such as Clone Manager Professional v9.2 (CloneSuit9), Clustal Omega and Jalview [30,31]. PCR amplification of *ompK*36 followed by Sanger sequencing was performed using previously described primers [15], when required for confirmation of whole genome sequencing results. The tridimensional structures of OmpK36 variants were assessed on the Phyre2 web portal for protein modelling, prediction and analysis, except for sequences of less than 30 amino acid residues, which are excluded by Phyre2 specifications.

Receiver operating characteristic (ROC) curves were built to visualize the performance ability of a disk diffusion assay using ertapenem (10 µg), meropenem (10 µg), imipenem (10 µg) and cefoxitin (30 µg) (Bio-Rad, Hercules, CA, USA), to detect OmpK36 functional alterations. In order to identify the optimal cut-off values to discriminate strains carrying functional alterations in this porin, a figure showing the evolution of sensitivity and 1-specificity in relation to the threshold changes was plotted for each of these antibiotics. This analysis was performed using Python programming language (Python Software Foundation. Python Language Reference, version 3.7., available at http://www.python.org, accessed on 8 March 2021, scikit-learn library) and visualized using matplotlib [32].

Genomes of all isolates studied in this work have been deposited in GenBank and their accession numbers are shown in Supplementary Table S1.

The present study was approved by the ethics committee of the Principality of Asturias.

## 4. Conclusions

In summary, we found that OmpK36 alterations could play an important role on the resistance to carbapenems of OXA-48-producing *K. pneumoniae*, consistent with the fact that this porin constitutes the main pathway of entrance of these drugs into the cell. Although our study only includes isolates recovered from a single hospital, they represent a great variety of *K. pneumoniae* clones, and different mutations in *ompK36* were found. As far as we know, our study represents the most extensive and complete molecular analysis of OXA-48-producing *K. pneumoniae* with alterations in the outer membrane porins. We have also demonstrated that these alterations do not affect the susceptibility to ceftazidime/avibactam, and thus this drug combination could be positioned as the choice therapy to combat the infections caused by this difficult to treat isolates. Finally, phenotypic detection of isolates carrying functional alterations on OmpK36 in clinical microbiology laboratories is important, and cefoxitin together with ertapenem/meropenem low inhibition zone diameters would strongly suggest alterations in this porin.

## Figures and Tables

**Table 1:** Microbiological features of OXA-48-producing *Klebsiella pneumoniae* isolates displaying high carbapenem resistance due to porin alterations.

Strain	ST	Sample	Previous Carbapenem Exposure (days) ^1^	ESBL Enzyme	FOX MIC/IZD ^2^	MER MIC/IZD ^2^	ERT MIC/IZD^2^	IPM vMIC/IZD ^2^	CAZ/AVI MIC	*ompK35*	*ompK36*
Kp_HUCA_Bac_5	326	Blood culture	Yes (10)	CTX-M-15	>16/6	>32/6	>4/6	>8/6	0.5	WT	::IS*5*
Kp_HUCA_Bac_7	326	Blood culture	No	CTX-M-15	>16/6	16/6	>4/6	>8/6	0.75	WT	::IS*5*
Kp_HUCA_Bac_10	326	Blood culture	Yes (21)	CTX-M-15	>16/6	>32/8	>4/6	>8/9	0.5	WT	ΔC201
Kp_HUCA_Bac_13	326	Blood culture	Yes (11)	CTX-M-15	>16/6	>32/9	>4/7	>8/11	0.75	WT	G259T
Kp_HUCA_Bac_18	326	Blood culture	No	CTX-M-15	>16/7	32/8	>4/6	>8/9	0.5	WT	ΔC201
Kp_HUCA_Bac_24	326	Blood culture	Yes (5)	CTX-M-15	>16/11	>32/6	>1/6	>8/9	0.5	::623T	::IS*5*
Kp_HUCA_Bac_64	16	Blood culture	Yes (5)	CTX-M-15	16/11	8/16	>4/16	8/18	0.5	WT	ΔA74
Kp_HUCA_Bac_65	147	Blood culture	No	SHV-12	≤8/15	8/16	>1/16	4/19	0.38	WT	::IS*5*
Kp_HUCA_Bac_76	405	Blood culture	Yes (7)	CTX-M-15	>16/12	>32/6	>1/6	>8/6	0.75	::623T	T612A
Kp_HUCA_Bac_85	273	Blood culture	Yes (19)	Non-ESBL	>16/6	>32/6	>1/6	>8/6	0.5	::623T	::29TG
Kp_HUCA_Bac_88	405	Blood culture	Yes (6)	Non-ESBL	16/18	8/16	>1/16	8/17	0.19	WT	::818ACAAAGCGCAGAACTTCGAACCTGGGCTTTGCAA
Kp_HUCA_Bac_90	101	Blood culture	Yes (2)	CTX-M-15	>16/6	24/6	>1/6	4/17	0.5	ΔG185	::403GACGGC
Kp_HUCA_2	326	Rectal swab	No	CTX-M-15	>16/6	>32/6	>1/6	>8/8	0.5	WT	G844T
Kp_HUCA_3	15	Sputum	No	CTX-M-15	>16/6	>32/6	>1/6	>8/14	0.75	::623T	::403GACGGC
Kp_HUCA_4	15	Urine	Yes (15)	CTX-M-15	>16/7	>32/6	>1/6	>8/6	0.75	WT	Δ(886)-184
Kp_HUCA_5	193	Urine	No	Non-ESBL	>16/8	16/12	>1/10	>8/12	0.5	WT	::IS*1*
Kp_HUCA_8	326	Rectal swab	Yes (4)	CTX-M-15	>16/6	32/6	>1/6	>8/9	0.5	WT	ΔA4

ST, sequence type; ESBL, extended-spectrum beta-lactamase; FOX, cefoxitin; MER, meropenem; ERT, ertapenem; IPM, imipenem; CAZ/AVI, ceftazidime/avibactam; MIC, minimal inhibitory concentration; IZD, inhibition zone diameter; WT, wild type.^1^ This column indicates if the patient received a carbapenem during hospital admission before the isolate was recovered, with the number of days receiving this drug shown in brackets.^2^ MIC are expressed in mg/L and IZD in mm.

## Data Availability

Data is contained within the article and/or supplemental materials. Genomes have been deposited in GenBank. Additional data are freely available on request from the corresponding author.

## Supplementary Materials

The following are available online at https://www.mdpi.com/article/10.3390/antibiotics10101174/s1, Figure S1: Tridimensional analysis, assessed by the Phyre2 web, of OmpK36 protein structures of OXA-48-producing *Klebsiella pneumoniae* isolates displaying high-level resistance to meropenem. This web was not able to create structures for Kp_HUCA_Bac_5, Kp_HUCA_Bac_24, Kp_HUCA_Bac_65, Kp_HUCA_Bac_85, Kp_HUCA_4 and Kp_HUCA_8 since the proteins predicted had less than 30 amino acids., Table S1: GenBank accession numbers of the genomes of OXA-48-producing Klebsiella pneumoniae isolates, Table S2: Microbiological features of OXA-48-producing Klebsiella pneumoniae isolates displaying meropenem MICs lower than 8 mg/L.
