# Supplementary material for: High-Level Carbapenem Resistance among OXA-48-Producing Klebsiella pneumoniae with Functional OmpK36 Alterations: Maintenance of Ceftazidime/Avibactam Susceptibility
Source: Antibiotics (Basel). 2021 Sep 27;10(10):1174. doi: 10.3390/antibiotics10101174 (PMC8532661; doi:10.3390/antibiotics10101174)
Supplement: Supplementary file 1 [file antibiotics-10-01174-s001.zip › FigureS1.pptx]

## Slide 1
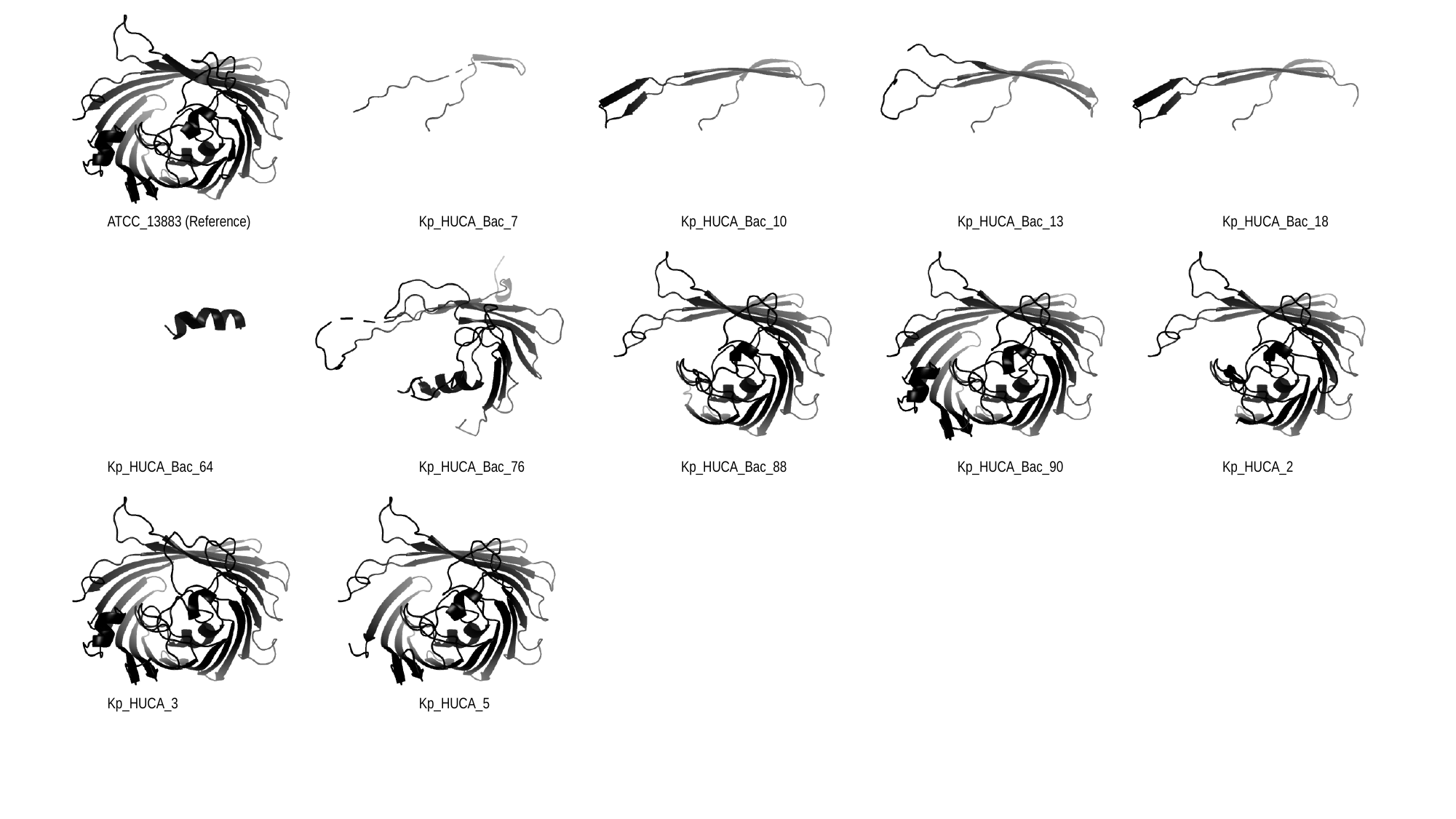

ATCC_13883 (Reference)
Kp_HUCA_Bac_7
Kp_HUCA_Bac_10
Kp_HUCA_Bac_13
Kp_HUCA_Bac_18
Kp_HUCA_Bac_64
Kp_HUCA_Bac_76
Kp_HUCA_Bac_88
Kp_HUCA_Bac_90
Kp_HUCA_2
Kp_HUCA_3
Kp_HUCA_5
