# Supplementary material for: High-Level Carbapenem Resistance among OXA-48-Producing Klebsiella pneumoniae with Functional OmpK36 Alterations: Maintenance of Ceftazidime/Avibactam Susceptibility
Source: Antibiotics (Basel). 2021 Sep 27;10(10):1174. doi: 10.3390/antibiotics10101174 (PMC8532661; doi:10.3390/antibiotics10101174)
Supplement: Supplementary file 1 [file antibiotics-10-01174-s001.zip › TableS1.docx]

| **TableS1:** GenBank accession numbers of the genomes of OXA-48-producing *Klebsiella* *pneumoniae* isolates | |
| --- | --- |
| Strain | Accession number |
| Kp_HUCA_Bac_1 | JAGLAK000000000 |
| Kp_HUCA_Bac_2 | JAGLAJ000000000 |
| Kp_HUCA_Bac_3 | JAGLAI000000000 |
| Kp_HUCA_Bac_4 | JAGLAH000000000 |
| Kp_HUCA_Bac_5 | JAGLAG000000000 |
| Kp_HUCA_Bac_6 | JAGLAF000000000 |
| Kp_HUCA_Bac_7 | JAGLAE000000000 |
| Kp_HUCA_Bac_8 | JAGLAD000000000 |
| Kp_HUCA_Bac_9 | JAGLAC000000000 |
| Kp_HUCA_Bac_10 | JAGLAB000000000 |
| Kp_HUCA_Bac_11 | JAGLAA000000000 |
| Kp_HUCA_Bac_13 | JAGKZZ000000000 |
| Kp_HUCA_Bac_15 | JAGKZY000000000 |
| Kp_HUCA_Bac_16 | JAGKZX000000000 |
| Kp_HUCA_Bac_17 | JAGKZW000000000 |
| Kp_HUCA_Bac_18 | JAGKZV000000000 |
| Kp_HUCA_Bac_19 | JAGKZU000000000 |
| Kp_HUCA_Bac_20 | JAGKZT000000000 |
| Kp_HUCA_Bac_21 | JAGKZS000000000 |
| Kp_HUCA_Bac_23 | JAGKZR000000000 |
| Kp_HUCA_Bac_24 | JAGKZQ000000000 |
| Kp_HUCA_Bac_28 | JAGKZP000000000 |
| Kp_HUCA_Bac_30 | JAGKZO000000000 |
| Kp_HUCA_Bac_31 | JAGKZN000000000 |
| Kp_HUCA_Bac_33 | JAGKZM000000000 |
| Kp_HUCA_Bac_34 | JAGKZL000000000 |
| Kp_HUCA_Bac_35 | JAGKZK000000000 |
| Kp_HUCA_Bac_36 | JAGKZJ000000000 |
| Kp_HUCA_Bac_37 | JAGKZI000000000 |
| Kp_HUCA_Bac_38 | JAGKZH000000000 |
| Kp_HUCA_Bac_39 | JAGKZG000000000 |
| Kp_HUCA_Bac_42 | JAGKZF000000000 |
| Kp_HUCA_Bac_43 | JAGKZE000000000 |
| Kp_HUCA_Bac_44 | JAGKZD000000000 |
| Kp_HUCA_Bac_45 | JAGKZC000000000 |
| Kp_HUCA_Bac_46 | JAGKZB000000000 |
| Kp_HUCA_Bac_47 | JAGKZA000000000 |
| Kp_HUCA_Bac_48 | JAGKYZ000000000 |
| Kp_HUCA_Bac_49 | JAGKYY000000000 |
| Kp_HUCA_Bac_50 | JAGKYX000000000 |
| Kp_HUCA_Bac_51 | JAGKYW000000000 |
| Kp_HUCA_Bac_52 | JAGKYV000000000 |
| Kp_HUCA_Bac_53 | JAGKYU000000000 |
| Kp_HUCA_Bac_54 | JAGKYT000000000 |
| Kp_HUCA_Bac_56 | JAGKYS000000000 |
| Kp_HUCA_Bac_57 | JAGKYR000000000 |
| Kp_HUCA_Bac_60 | JAGKYP000000000 |
| Kp_HUCA_Bac_62 | JAGKYO000000000 |
| Kp_HUCA_Bac_63 | JAGKYN000000000 |
| Kp_HUCA_Bac_64 | JAGKYM000000000 |
| Kp_HUCA_Bac_65 | JAGKYL000000000 |
| Kp_HUCA_Bac_66 | JAGKYK000000000 |
| Kp_HUCA_Bac_67 | JAGKYJ000000000 |
| Kp_HUCA_Bac_68 | JAGKYI000000000 |
| Kp_HUCA_Bac_69 | JAGKYH000000000 |
| Kp_HUCA_Bac_71 | JAGKYG000000000 |
| Kp_HUCA_Bac_72 | JAGKYF000000000 |
| Kp_HUCA_Bac_73 | JAGKYE000000000 |
| Kp_HUCA_Bac_74 | JAGKYD000000000 |
| Kp_HUCA_Bac_75 | JAGKYC000000000 |
| Kp_HUCA_Bac_76 | JAGKYB000000000 |
| Kp_HUCA_Bac_78 | JAGKYA000000000 |
| Kp_HUCA_Bac_79 | JAGKXZ000000000 |
| Kp_HUCA_Bac_80 | JAGKXY000000000 |
| Kp_HUCA_Bac_81 | JAGKXX000000000 |
| Kp_HUCA_Bac_82 | JAGKXW000000000 |
| Kp_HUCA_Bac_83 | JAGKXV000000000 |
| Kp_HUCA_Bac_84 | JAGKXU000000000 |
| Kp_HUCA_Bac_85 | JAGKXT000000000 |
| Kp_HUCA_Bac_88 | JAGKXS000000000 |
| Kp_HUCA_Bac_89 | JAGKXR000000000 |
| Kp_HUCA_Bac_90 | JAGKXQ000000000 |
| Kp_HUCA_Bac_92 | JAGKXP000000000 |
| Kp_HUCA_Bac_93 | JAGKXO000000000 |
| Kp_HUCA_Bac_94 | JAGKXN000000000 |
| Kp_HUCA_Bac_95 | JAGKXM000000000 |
| Kp_HUCA_2 | JAGKXK000000000 |
| Kp_HUCA_3 | JAGKXJ000000000 |
| Kp_HUCA_4 | JAGKXI000000000 |
| Kp_HUCA_5 | JAGKXH000000000 |
| Kp_HUCA_8 | JAGKXE000000000 |
