# Supplementary material for: High-Level Carbapenem Resistance among OXA-48-Producing Klebsiella pneumoniae with Functional OmpK36 Alterations: Maintenance of Ceftazidime/Avibactam Susceptibility
Source: Antibiotics (Basel). 2021 Sep 27;10(10):1174. doi: 10.3390/antibiotics10101174 (PMC8532661; doi:10.3390/antibiotics10101174)
Supplement: Supplementary file 1 [file antibiotics-10-01174-s001.zip › TableS2.docx]

**Table S2:** Microbiological features of OXA-48-producing Klebsiella pneumoniae isolates displaying meropenem MICs lower than 8mg/L.

| Strain | ST | Sample | ESBL  enzyme | FOX | MER | ERT | IPM | *ompK35* |
| --- | --- | --- | --- | --- | --- | --- | --- | --- |
|  |  |  |  | MIC/IZD^1^ | MIC/IZD^1^ | MIC/IZD^1^ | MIC/IZD^1^ |  |
| Kp_HUCA_Bac_1 | 15 | Blood culture | Non-ESBL | <=8/18 | 2/18 | 2/17 | <=1/19 | ::IS*1* |
| Kp_HUCA_Bac_2 | 104 | Blood culture | CTX-M-15/SHV-71 | <=8/23 | 0,125/19 | 1/19 | <=1/19 | WT |
| Kp_HUCA_Bac_3 | 15 | Blood culture | CTX-M-15 | <=8/22 | 0,75/20 | 2/19 | <=1/20 | WT |
| Kp_HUCA_Bac_4 | 104 | Blood culture | CTX-M-15/SHV-71 | <=8/23 | 0,5/20 | 1/21 | <=1/20 | WT |
| Kp_HUCA_Bac_6 | 326 | Blood culture | CTX-M-15 | 16/15 | 1/18 | 4/18 | <=1/16 | WT |
| Kp_HUCA_Bac_8 | 326 | Blood culture | CTX-M-15 | <=8/19 | 2/17 | 4/17 | 2/16 | WT |
| Kp_HUCA_Bac_9 | 326 | Blood culture | CTX-M-15 | 16/15 | 1/20 | 4/18 | <=1/18 | WT |
| Kp_HUCA_Bac_11 | 147 | Blood culture | SHV-12 | <=8/20 | 2/18 | 4/17 | <=1/17 | WT |
| Kp_HUCA_Bac_15 | 147 | Blood culture | SHV-12 | <=8/20 | 4/20 | 2/19 | <=1/21 | WT |
| Kp_HUCA_Bac_16 | 326 | Blood culture | CTX-M-15 | 16/17 | 0,25/21 | 4/18 | <=1/22 | WT |
| Kp_HUCA_Bac_17 | 405 | Blood culture | Non-ESBL | <=8/21 | 1,5/20 | 4/18 | <=1/22 | WT |
| Kp_HUCA_Bac_19 | 326 | Blood culture | CTX-M-15 | 16/16 | 0,75/22 | 4/19 | 2/23 | WT |
| Kp_HUCA_Bac_20 | 353 | Blood culture | Non-ESBL | <=8/24 | 0,25/21 | 1/20 | <=1/20 | WT |
| Kp_HUCA_Bac_21 | 326 | Blood culture | CTX-M-15 | <=8/23 | 1/22 | 1/21 | <=1/22 | WT |
| Kp_HUCA_Bac_23 | 147 | Blood culture | SHV-12 | <=8/21 | 0,38/18 | 2/17 | <=1/18 | ::IS*1* |
| Kp_HUCA_Bac_28 | 147 | Blood culture | SHV-12 | <=8/20 | 3/20 | 4/18 | <=1/22 | WT |
| Kp_HUCA_Bac_30 | 147 | Blood culture | SHV-12 | <=8/24 | 6/19 | 1/19 | 2/19 | WT |
| Kp_HUCA_Bac_31 | 326 | Blood culture | CTX-M-15 | 16/17 | 0,25/20 | 2/19 | <=1/22 | WT |
| Kp_HUCA_Bac_33 | 16 | Blood culture | CTX-M-15 | <=8/20 | 2/21 | 4/20 | 2/22 | WT |
| Kp_HUCA_Bac_34 | 405 | Blood culture | CTX-M-15 | <=8/23 | 1,5/21 | 4/20 | 2/20 | WT |
| Kp_HUCA_Bac_35 | 405 | Blood culture | CTX-M-15 | 16/14 | 2/20 | 4/19 | <=1/23 | WT |
| Kp_HUCA_Bac_36 | 405 | Blood culture | CTX-M-15 | <=8/23 | 1,5/19 | 1/19 | <=1/18 | WT |
| Kp_HUCA_Bac_37 | 15 | Blood culture | Non-ESBL | 16/22 | 1/19 | >1/18 | 2/19 | G890A |
| Kp_HUCA_Bac_38 | 16 | Blood culture | Non-ESBL | <=8/19 | 2/18 | 2/17 | <=1/18 | WT |
| Kp_HUCA_Bac_39 | 405 | Blood culture | CTX-M-15 | <=8/22 | 2/18 | 1/17 | <=1/18 | WT |
| Kp_HUCA_Bac_42 | 16 | Blood culture | Non-ESBL | <=8/19 | 0,38/20 | >1/19 | <=1/21 | WT |
| Kp_HUCA_Bac_43 | 147 | Blood culture | SHV-12 | <=8/22 | 3/22 | 2/20 | 2/21 | WT |
| Kp_HUCA_Bac_44 | 405 | Blood culture | CTX-M-15 | <=8/26 | 2/22 | 2/21 | 2/21 | WT |
| Kp_HUCA_Bac_45 | 405 | Blood culture | CTX-M-15 | <=8/25 | 1,5/22 | 1/22 | <=1/20 | WT |
| Kp_HUCA_Bac_46 | 16 | Blood culture | CTX-M-15 | <=8/20 | 0,19/23 | 2/21 | <=1/24 | WT |
| Kp_HUCA_Bac_47 | 405 | Blood culture | CTX-M-15 | <=8/24 | 1/21 | 2/21 | 2/21 | WT |
| Kp_HUCA_Bac_48 | 326 | Blood culture | CTX-M-15 | 16/17 | 0,38/22 | 1/20 | <=1/24 | WT |
| Kp_HUCA_Bac_49 | 147 | Blood culture | SHV-12 | <=8/20 | 0,5/21 | 2/19 | <=1/22 | WT |
| Kp_HUCA_Bac_50 | 15 | Blood culture | Non-ESBL | <=8/20 | 3/18 | 2/18 | 2/18 | WT |
| Kp_HUCA_Bac_51 | 326 | Blood culture | CTX-M-15 | <=8/25 | 0,75/24 | 4/24 | 2/22 | WT |
| Kp_HUCA_Bac_52 | 147 | Blood culture | SHV-12 | <=8/20 | 0,125/23 | 2/20 | <=1/24 | WT |
| Kp_HUCA_Bac_53 | 326 | Blood culture | CTX-M-15 | <=8/20 | 0,25/22 | 1/21 | 1/24 | WT |
| Kp_HUCA_Bac_54 | 326 | Blood culture | CTX-M-15 | >16/11 | 0,19/24 | 2/21 | <=1/25 | WT |
| Kp_HUCA_Bac_56 | 405 | Blood culture | CTX-M-15 | <=8/26 | 0,19/24 | 2/23 | 2/23 | WT |
| Kp_HUCA_Bac_57 | 323 | Blood culture | Non-ESBL | <=8/24 | 1/20 | 2/20 | 2/19 | WT |
| Kp_HUCA_Bac_60 | 326 | Blood culture | CTX-M-15 | 16/18 | 0,75/22 | 2/19 | <=1/22 | WT |
| Kp_HUCA_Bac_62 | 405 | Blood culture | CTX-M-15 | <=8/21 | 1,5/22 | 2/20 | <=1/20 | WT |
| Kp_HUCA_Bac_63 | 147 | Blood culture | SHV-12 | <=8/21 | 1/21 | 2/19 | 2/22 | WT |
| Kp_HUCA_Bac_66 | 147 | Blood culture | SHV-12 | <=8/25 | 1/23 | >1/22 | 2/24 | WT |
| Kp_HUCA_Bac_67 | 147 | Blood culture | SHV-12 | <=8/22 | 4/19 | 2/18 | 2/18 | WT |
| Kp_HUCA_Bac_68 | 881 | Blood culture | Non-ESBL | <=8/25 | 3/23 | 2/22 | <=1/25 | WT |
| Kp_HUCA_Bac_69 | 326 | Blood culture | CTX-M-15 | 16/15 | 0,38/23 | 4/20 | <=1/23 | WT |
| Kp_HUCA_Bac_71 | 147 | Blood culture | SHV-12 | 16/14 | 0,75/20 | >1/17 | <=1/21 | WT |
| Kp_HUCA_Bac_72 | 15 | Blood culture | CTX-M-15 | <=8/21 | 0,19/25 | >1/22 | <=1/26 | WT |
| Kp_HUCA_Bac_73 | 15 | Blood culture | Non-ESBL | <=8/20 | 0,25/26 | >1/24 | <=1/23 | ::IS*1* |
| Kp_HUCA_Bac_74 | 405 | Blood culture | CTX-M-15 | 16/26 | 0,38/25 | 1/24 | <=1/29 | WT |
| Kp_HUCA_Bac_75 | 15 | Blood culture | CTX-M-15 | <=8/21 | 0,38/24 | >1/20 | <=1/25 | WT |
| Kp_HUCA_Bac_78 | 147 | Blood culture | SHV-12 | <=8/22 | 1/19 | >1/17 | 4/20 | ΔG575 |
| Kp_HUCA_Bac_79 | 147 | Blood culture | SHV-12 | <=8/20 | 0,19/23 | 2/20 | 2/25 | WT |
| Kp_HUCA_Bac_80 | 326 | Blood culture | CTX-M-15 | <=8/25 | 0,25/25 | >1/23 | <=1/25 | WT |
| Kp_HUCA_Bac_81 | 15 | Blood culture | CTX-M-15 | <=8/21 | 0,38/22 | >1/20 | <=1/23 | WT |
| Kp_HUCA_Bac_82 | 485 | Blood culture | Non-ESBL | <=8/23 | 0,38/21 | >1/20 | 2/18 | WT |
| Kp_HUCA_Bac_83 | 147 | Blood culture | SHV-12 | <=8/20 | 2/21 | >1/20 | 2/21 | WT |
| Kp_HUCA_Bac_84 | 567 | Blood culture | Non-ESBL | <=8/26 | 0,125/24 | >1/22 | <=1/22 | WT |
| Kp_HUCA_Bac_89 | 147 | Blood culture | SHV-12 | <=8/19 | 2/23 | >1/21 | 2/25 | WT |
| Kp_HUCA_Bac_92 | 147 | Blood culture | SHV-12 | >16/19 | 0,25/21 | >1/20 | 8/22 | WT |
| Kp_HUCA_Bac_93 | 307 | Blood culture | CTX-M-15 | <=8/23 | 1/21 | 1/20 | <=1/22 | WT |
| Kp_HUCA_Bac_94 | 147 | Blood culture | SHV-12 | <=8/18 | 1/21 | >1/19 | <=1/22 | WT |
| Kp_HUCA_Bac_95 | 147 | Blood culture | SHV-12 | <=8/21 | 1/22 | >1/20 | 2/22 | WT |

ST, sequence type; ESBL, extended-spectrum β-lactamase; FOX, cefoxitin; MER, meropenem; ERT, ertapenem; IPM, imipenem; MIC, minimal inhibitory concentration; IZD, inhibition zone diameter; WT, wild type; NA, not applicable; ^1^MIC are expressed in mg/L and IZD in mm. All isolates were wild type for *OmpK36*.
